# Diagnostic accuracy of artificial intelligence for spinopelvic parameters in standing total spine X-ray and limitations after fusion surgery

**DOI:** 10.1007/s00256-026-05289-x

**Published:** 2026-07-04

**Authors:** Tobias Winkler, Thilo Khakzad, Matthias Pumberger, Friederike Schömig, Matthias Becher, Torsten Diekhoff

**Affiliations:** 1 Department of Radiology, Charité - Universitätsmedizin Berlin, Campus Mitte, Humboldt-Universität Zu Berlin, Freie Universität Berlin, Charitéplatz 1, 10117 Berlin, Germany; 2 Center for Musculoskeletal Surgery, Charité - Universitätsmedizin Berlin, Campus Mitte, Humboldt-Universität Zu Berlin, Freie Universität Berlin, Charitéplatz 1, 10117 Berlin, Germany; 3 Institute of Biometry and Clinical Epidemiology, Charité - Universitätsmedizin Berlin, Campus Mitte, Humboldt-Universität Zu Berlin, Freie Universität Berlin, Charitéplatz 1, 10117 Berlin, Germany; 4 Department of Radiology, Immanuel Clinic Ruedersdorf, Brandenburg Medical School, Ruedersdorf Bei Berlin, Germany

**Keywords:** Artificial intelligence, Spine, Radiography, Diagnostic imaging, Observer variation

## Abstract

**Objective:**

To evaluate the accuracy of a commercial deep learning algorithm in measuring spinopelvic parameters on full spine radiographs.

**Materials and methods:**

This retrospective study analyzed total spine X-rays from a clinical cohort, assessing spinopelvic parameters (coronal Cobb angle, thoracic kyphosis, lumbar lordosis, pelvic incidence, sagittal vertical axis). Measurements were performed by clinical radiologists, a trained research reader serving as ground truth, and a commercial AI software. Inter-rater reliability was determined via intraclass correlation coefficients between an orthopedic surgeon and the trained reader (*n* = 50 images). Performance of both the AI and radiologists was statistically compared to the ground truth using mean absolute error, intraclass correlation coefficients, the Spearman correlation, and diagnostic accuracy metrics (sensitivity, specificity, predictive values).

**Results:**

Four hundred ninety-five radiographs were analyzed. Inter-rater reliability between human raters, assessed via intraclass correlation coefficients, was excellent (0.94–1). Agreement between the algorithm and ground truth, also assessed via intraclass correlation coefficients, was good to excellent (0.79–0.99), except for lumbar lordosis (0.68, moderate). Mean absolute errors were lowest for coronal Cobb angles (4,6°; 95% confidence interval = 3.6–5.5°) and highest for lumbar lordosis (8.1°; 95% confidence interval = 6.9–9.3°). The Spearman rank correlations ranged from 0.74 to 0.99, and sensitivity was moderate to excellent (72.6–94.8). These results closely matched those obtained when comparing radiologists to ground truth. In a subgroup of patients with prior spinal fusion surgery, the correlation between the algorithm predictions and the ground truth was reduced, and measurement deviations were higher compared to non-instrumented patients.

**Conclusion:**

The commercial AI software predicted most spinopelvic parameters with good reliability and accuracy, coinciding with radiologists in clinical practice, however, showed limitations in patients with spinal instrumentation.

**Supplementary Information:**

The online version contains supplementary material available at https://doi.org/10.1007/s00256-026-05289-x.

## Introduction

For the comprehensive assessment of spinal alignment and balance, several measures were introduced on total spine X-rays which became clinical standard for the treatment of many spinal conditions like degenerative pathologies and adult spinal deformity [1]. However, the routine performance of these measurements contributes to the increasing workload radiologists’ phase, exacerbated due to a persistent rise in the volume of radiological imaging examinations [2]. At the same time, numerous studies have shown a correlation between an increased caseload of radiologists and a subsequent decline in interpretive accuracy [3, 4]. Consequently, there exists a pressing need for an automated, standardized, rapid, and precise solution to maintain accurate measurements despite rising case numbers. Recent advancements in deep learning (DL) and artificial intelligence (AI) methodologies offer promising solutions for standardized and efficient image evaluation for various tasks [5–8]. While prior studies already explored AI-based measurements of spinopelvic parameters, these investigations focused primarily on validating algorithms against curated datasets using a single rater or a small group of raters as ground truth [9, 10]. Therefore, there is a critical gap in evaluating AI performance under real-world conditions, where measurements are influenced by inter-rater variability among radiologists with varying experience levels [11]. Our study externally validates a commercially available AI software for measuring spinopelvic parameters by comparing its performance not only to a trained research reader but also to clinical radiologists’ routine measurements.

We hypothesized that the AI system would demonstrate high measurement accuracy relative to real-world assessments, thereby providing a reliable and efficient method for the assessment of EOS total spine images.

## Methods

### Ethics approval

Institutional Review Board approval for this study was obtained under protocol EA2/034/24. The research was conducted in strict adherence to established ethical standards, including those stipulated in the World Medical Association Declaration of Helsinki.

### Patient selection

**Fig. 1:**
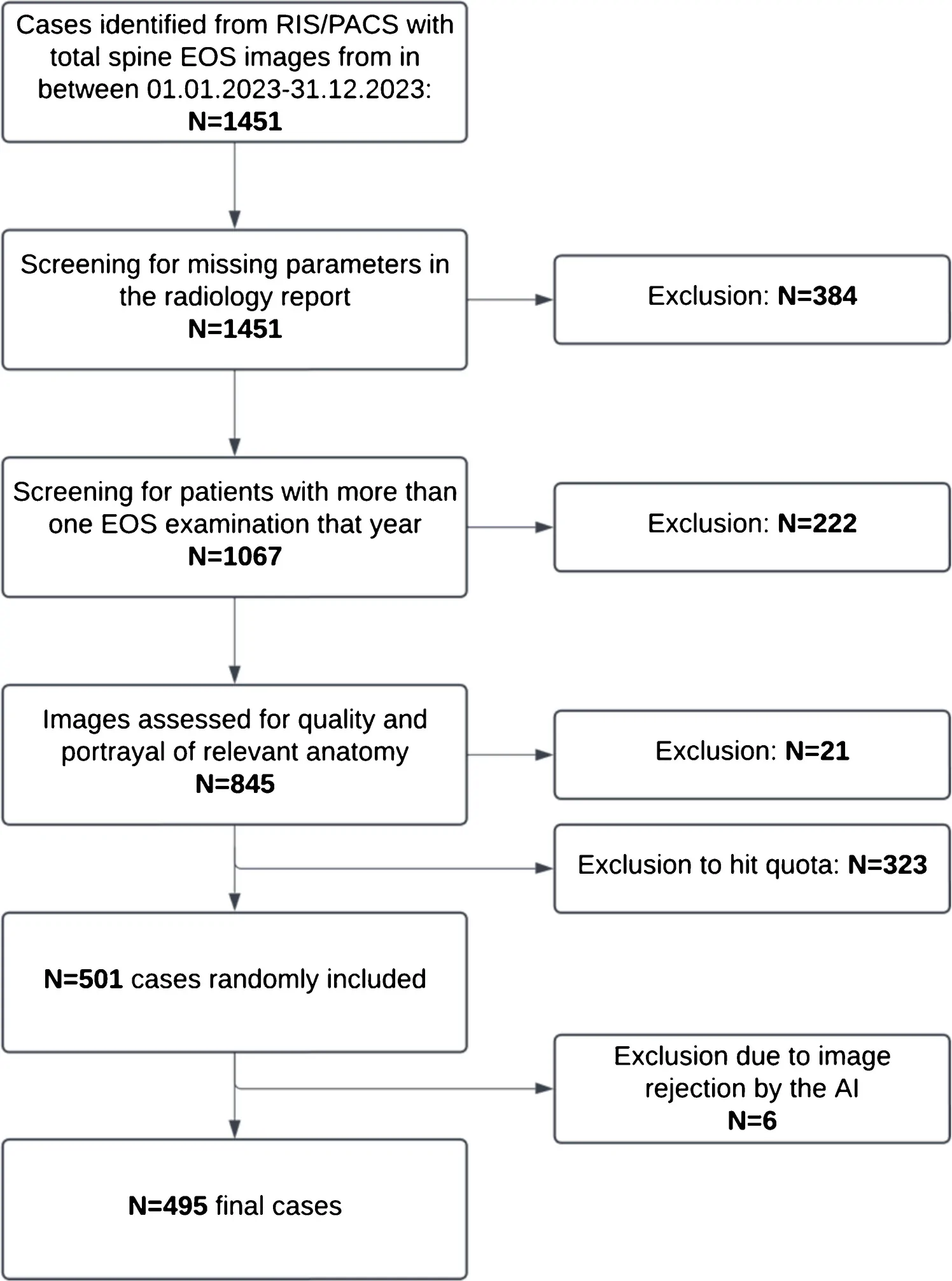
Flowchart of patient inclusion

In this retrospective validation study, total spine X-rays were obtained from a university hospital (Charité – Universitätsmedizin Berlin) utilizing the radiology information system and the picture archive and communication system (RIS/PACS). The inclusion criteria were delineated as follows: (1) Patients had undergone standing total spine radiography imaging on a bi-planar stereographic X-ray system (EOS imaging, Paris, France) during the period from January 1, 2023, to December 31, 2023. (2) Both anteroposterior and lateral views must be available. (3) A structured report encompassing all designated measurements must be available for these examinations. Examinations that lacked representation of the requisite anatomy (i.e., C2 to S1 for anteroposterior and C2 to femoral heads for lateral views) or exhibited significant artifacts that obstructed accurate measurements were excluded from the study. In cases where patients had multiple examinations within the specified timeframe, only the first image set was deemed eligible for analysis to avoid mix-ups and enable reproducibility of the dataset. Out of the remaining cohort, patients were included randomly until the aimed quota was reached. The flowchart of patient inclusion is shown in Fig. 1.

### Imaging

The bi-planar radiographs were performed in a free-standing position. A tube voltage (kV) of 83–100 kVp (ap) and 102–110 kVp (lateral) with a tube current (mA) of 200–250 (ap) and 200–320 (lateral), depending on the patients’ body mass index, was used for capturing the images. The imaging protocol was optimized for high-resolution low-dose acquisition using the µDose feature.

### Report assessment

Measurements in clinical routine were performed by a radiology resident or a board-certified radiologist. Each radiology report was signed by a board-certified radiologist. From each report, the following parameters were extracted: coronal Cobb angle (CC), thoracic kyphosis (TK), lumbar lordosis (LL), pelvic incidence (PI), and sagittal vertical axis (SVA). Presence of metal, especially spinal fusion, degeneration, and vertebral compression fractures was also assessed.

### Image processing

Spinal angle measurements were performed using the commercial AI-based MSK measurement software Gleamer, Paris, France, which was installed on an in-house server. This CE-marked, fully automated radiological image-processing software for conventional and EOS radiographs uses several convolutional neural networks for pose estimation and anatomical key-point detection, including top-down Mask R-CNN/Detectron2-derived, lightweight HRNet, and bottom-up architectures. Predicted key points are assigned confidence scores from 0 to 100, and only key points above a threshold of 50 are used for measurement calculation. Further proprietary details, including model weights and training hyperparameters, were not disclosed by the manufacturer. Subsequently, the images underwent processing, and measurements, along with a detailed report, were transferred into the PACS system from which the parameters were extracted.

### Reference standard

Reader 1 (R1), a medical research student, received training from a senior consultant radiologist specializing in musculoskeletal imaging with 15 years of experience, to measure the corresponding angles and parameters. A validation test group consisting of 20 examinations, which were not included in the final analysis, was utilized for this purpose. Subsequently, R1 evaluated the anonymized images of the cohort, blind to clinical data, AI measurements, and the content of the radiology report. For patients with a complete set of measurements from both R1 and the AI, a subset of 50 randomly selected patients was assessed by an orthopedic surgeon (R2) with 8 years of experience using the same blinded methodology to calculate the inter-rater reliability. The measurements by R1 served as reference standard for this analysis. Detailed descriptions of how these parameters were assessed can be found in the supplementary methods section of the electronic supplement.

### Reference values

A CC was regarded as negative if it exhibited values below 10° [12]. Normal values for TK were established at 30–50° and LL at 50–60° (from L1 to S1) and 30–50° (from L1 to L5) [13]. PI was deemed normal if within the range of 40–55°, while SVA was considered normal if between +50 and 0 mm [14]. All other values were classified as atypical.

### Statistical analysis

All statistical tests have been performed using the IBM SPSS statistics program (version 29.0.0.0 (241) and 29.0.2.0 (20)).

CC values below 10° were treated as 0° (negative), and only the highest CC from each examination was included in the analysis. Examinations that lacked data from the AI processing were omitted from the comparative analysis of measurements that were not conducted.

Descriptive statistics were used to categorize the study cohort: age as mean and standard deviation, sex as absolute frequencies and percentages, and other categorical variables (spinal instrumentation, fractures, degenerative changes) as absolute frequencies. The Kolmogorov-Smirnov tests were utilized to assess the normality of our dataset for each measurement. We evaluated the precision and reliability of the AI and the report by determining the mean absolute error (MAE) and median absolute error (MedAE) compared to the reference standard. A Wilcoxon signed-rank test (WSRT) was performed to evaluate whether these measuring differences were statistically significant (*p* < 0.05). Sensitivity, specificity, negative predictive value (NPV), and positive predictive value (PPV) were assessed to classify pathological and healthy parameters according to the above-mentioned reference values. Evaluation of these parameters was done as follows: < 60 as poor, > 60–70 as fair, > 70–80 as moderate, > 80–90 as good, and > 90 as excellent. 95% confidence intervals for MAEs were calculated using a bootstrap approach with 1000 resamples, while those for sensitivity and specificity were derived via profile likelihood estimation. The inter-rater reliability was tested by intraclass correlation coefficient (ICC) estimates and their 95% confidence intervals were calculated based on a single-measure, absolute agreement, two-way mixed (R1 vs. R2; R1 vs. AI) or two-way random (R1 vs. report; AI vs. report) effects model, in accordance with Koo et al. [15]. We categorized the ICC values as follows: < 0.5 as poor, 0.5–0.75 as moderate, 0.75–0.9 as good, and 0.9–1 as excellent reliability [15]. Additionally, the correlation between the measurements of the AI and the report compared to the ground truth was tested and visualized using the Spearman correlation test (Spearman’s rho: *ρ*) and Bland-Altman plots. A subgroup analysis was performed for patients with spinal instrumentation, degeneration and fractures as stated in the radiology report.

## Results

### Patient selection

Detailed demographics of the total population and the sub populations, consisting of the patients that received measurements for each parameter from all three raters (R1, AI, report), are shown in Table 1.

**Table 1:** Patient characteristics

	Total	CC	TK	LL	PI	SVA
Patients, *n*	495	434	293	320	306	308
*Age*						
Mean ± SD	50 ± 23.7	49.3 ± 23.4	49.7 ± 23.1	49.4 ± 23.0	46.9 ± 23.3	47.1 ± 23.4
Range	5–94	5–89	5–88	5–94	5–88	5–94
*Sex*						
Women, *n*	284 (57.4%)	249 (57,4%)	173 (59.0%)	200 (62.5%)	189 (61.8%)	192 (62.3%)
Men, *n*	211 (42.6%)	185 (42,6%)	120 (40.1%)	120 (37.5%)	117 (38.2%)	116 (37.7%)
Instrumentations	154	118	80	94	71	73
Fractures	61	48	35	32	31	32
Degeneration	276	239	163	177	159	162

The demographics and imaging findings of the overall cohort, along with those of the sub-cohorts with corresponding completed measurements, are presented. *SD*, standard deviation; *CC*, coronal Cobb angle; *TK*, thoracic kyphosis; *LL*, lumbar lordosis; *PI*, pelvic incidence; *SVA*, sagittal vertical axis

### Image post-processing

**Fig. 2:**
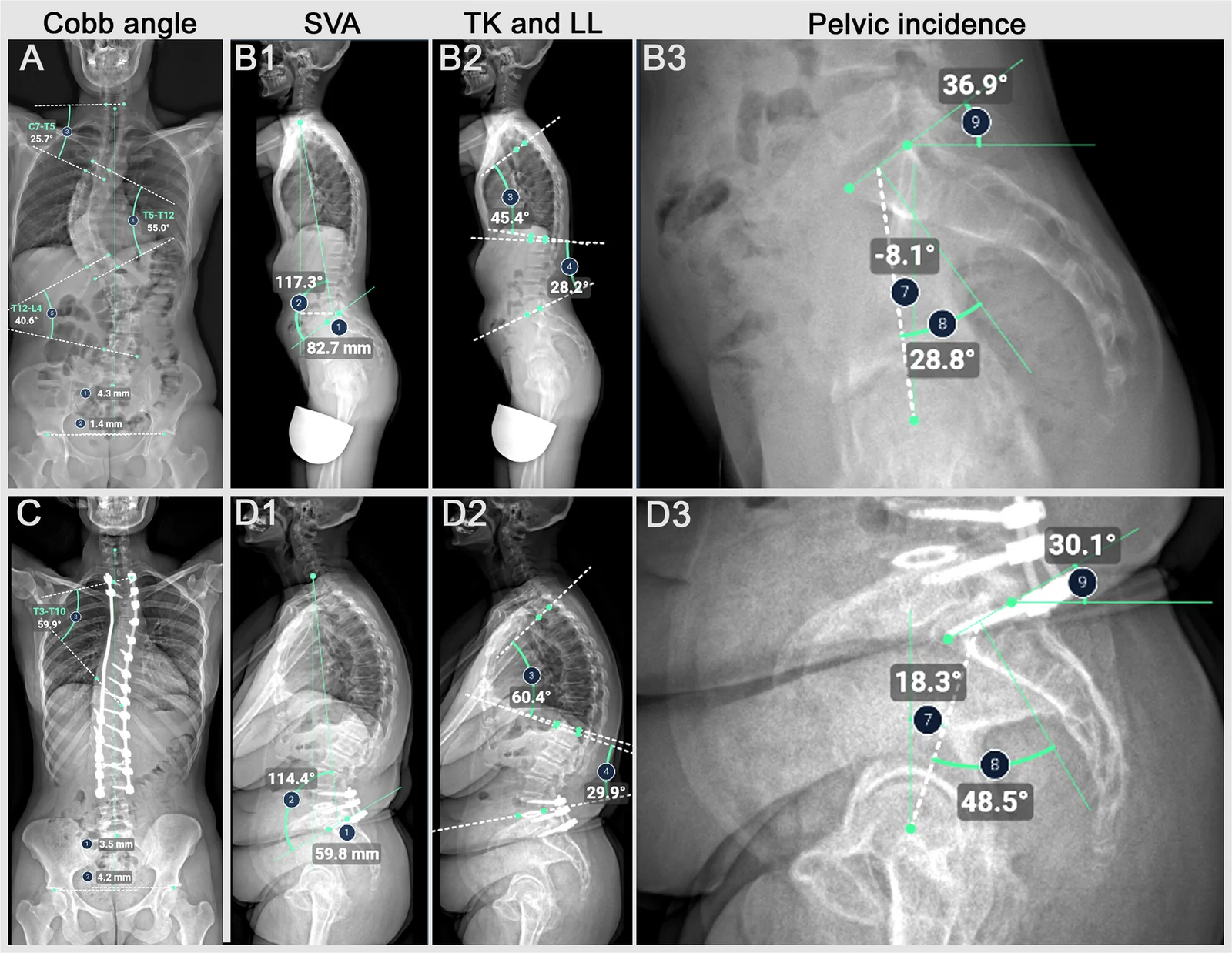
Examples of successful measurements (**A**, **B**) and unsuccessful measurements (**C**, **D**) of the tested artificial intelligence algorithm. Measurements for (**A**) and (**B**) are conducted with high precision. (**C**) A patient who has undergone corrective spinal fusion surgery, where challenges in identifying anatomical landmarks lead to an inaccurately elevated Cobb angle. (**D**) A patient with posterior lumbar spinal fusion. The LL is underestimated due to the selection of the endplate of L4 instead of L5. SVA is mildly overestimated, and PI measurements are influenced by the placement of screws in S1. SVA, sagittal vertical axis; TK, thoracic kyphosis; LL, lumbar lordosis; PI, pelvic incidence

Complete measurements were performed by the AI system in 210 (42.4%) cases. For 434 (87.7%) examinations, AI reported the CC, 293 (59.2%) the TK, 320 (64.6%) the LL, 306 (61.8%) the PI, and 308 (62.2%) the SVA. Examples of the AI measurements are shown in Fig. 2.

### Absolute measurements

The measurement results of R1, AI, and the report can be found in Table 2. While most measurements show very similar results across all raters, the most notable difference can be seen in the L1–L5 measurements, where the mean measurement value is 6.3° lower for the AI when compared to R1. Between AI and report, the biggest difference is found within the SVA measurements where the report exhibits a lower mean by 5.3°.

**Table 2:** Results of the measurements and correlation analyses

	Median measurement values (IQR) in °			Spearman correlation (*ρ*)	
	R1	AI	Report	AI	Report
CC	14.9 (0.0; 24.0)	14.8 (10.1; 24.5)	0.0 (0.0; 23.0)	0.82	0.83
*TK*	36.6 (27.5; 44.1)	37.1 (28.9; 46.6)	36.0 (27.0; 43.0)	0.84	0.87
*L1–L5*	39.9 (29.3; 49.9)	34.2 (21.0; 44.8)	-	0.74	-
*L1–S1*	51.8 (40.8; 61.1)	-	48.0 (36.0; 58.0)	-	0.82
*PI*	49.2 (41.8; 58.3)	51.2 (43.6; 61.4)	50.0 (42.0; 60.0)	0.88	0.81
*SVA*	11.0 (−17.8; 44.4)	11.1 (−17.3; 50.7)	7.0 (−20.0; 40.0)	0.99	0.98

The mean measurement values of each rater for all assessed parameters are shown, along with the comparison between AI and report regarding their Spearman’s rho (*ρ*) correlation coefficient to ground truth (R1). *ρ*, Spearman’s rho; *IQR*, interquartile range; *CC*, coronal Cobb angle; *TK*, thoracic kyphosis; L1–L5 and L1–S1, lumbar lordosis measurements; *PI*, pelvic incidence; *SVA*, sagittal vertical axis

### Comparative statistics

The original crosstabulations for these analyses can be found in Supplementary Tables S1–S10. The results of the Spearman correlation analysis are shown in Table 2; the ICC values are listed in Table 3.

**Table 3:** Results of the agreement analyses

	ICC (95% CI)			MAE in ° (95% CI)		WSRT
	R1 vs R2	R1 vs AI	R1 vs report	AI	Report	*p*-value
CC	0.99 (0.98; 1)	0.84 (0.82; 0.87)	0.88 (0.78; 0.92)	5.6 (4.9; 6.2)	5.2 (4.5; 5.9)	0.817
*TK*	0.96 (0.93; 0.98)	0.83 (0.79; 0.86)	0.87 (0.84; 0.90)	5.6 (4.9; 6.2)	4.7 (4.2; 5.4)	0.009
*L1–L5*	0.97 (0.95; 0.98)	0.68 (0.48; 0.79)	-	8.1 (6.9; 9.3)	-	0.035
*L1–S1*	0.94 (0.90; 0.97)	-	0.79 (0.70; 0.85)	-	6.0 (5.2; 6.9)	
*PI*	0.96 (0.92; 0.98)	0.79 (0.73; 0.83)	0.80 (0.75; 0.84)	5.2 (4.4; 6.1)	5.7 (5.0; 6.5)	0.069
*SVA*	1 (0.99; 1)	0.99 (0.98; 0.99)	0.98 (0.97; 0.99)	4.8 (4.1; 5.5)	6.1 (5.4; 7.0)	< 0.001

Agreement of R1 with R2 and AI assessed using two-way mixed-effects model with absolute agreement for single rater. Agreement of R1 with report assessed using two-way random-effects model with absolute agreement for single rater. Measuring deviation from ground truth assessed by MAE with significance evaluated by WSRT. *CI*, confidence interval; *ICC*, intraclass correlation coefficient; *MAE*, mean average error; *WSRT*, Wilcoxon signed-rank test; *CC*, coronal Cobb angle; *TK*, thoracic kyphosis; *L1–L5 and L1–S1*, lumbar lordosis measurements; *PI*, pelvic incidence; *SVA*, sagittal vertical axis

The inter-rater agreement between R1 and R2 was excellent (ICC < 0.9) for all measurements. The MAE was below 5° for all measurements except for LL, measured from L1 to S1, for which it was 5.22° ± 4.2°.

The Spearman correlation to the R1 measurements showed higher correlation with the report for CC, TK, and LL and higher correlation for the AI for PI and SVA measurements. The Bland-Altman plots (Supplementary Fig. S1) revealed lower mean biases between R1 and the report for TK, LL, and PI, whereas biases for CC and SVA were lower between R1 and AI. The 95% limits of agreement were comparable across most parameters. Both the AI and the report showed good ICCs for TK and PI measurements and excellent ICCs for SVA measurements. For CC measurements, the AI demonstrated a good, and the report an excellent ICC. LL measurements incorporated the lowest ICC for AI and the report with 0.68 (moderate) for the L1–L5 measurement by the AI and 0.79 (good) for the L1–S1 measurement in the report. Corresponding scatter plots for visualization are presented in Supplementary Fig. S2. According to the WSRT, significant measuring differences between AI and report were present in the TK (*p* = 0.009), LL (*p* = 0.035), and SVA (*p* < 0.001) measurements. The aberration of the MAE values of these parameters was 0.9° for TK with report < AI, 2.1° for LL with report < AI, and 1.3° for SVA with AI < report. Across all analyses, MedAE values were consistently lower than MAE values. The MedAE values for each parameter are provided in Supplementary Table S11.

### Diagnostic accuracy

The diagnostic accuracy of the report and the AI is presented in Table 4. The sensitivity analysis showed moderate to excellent results by the AI (72.6–94.8). Similar results were seen between AI and the report in the TK and LL measurements, with moderate (TK) and good (LL) results. In comparison, sensitivity was higher for measurements by the AI for CC (excellent compared to fair), PI (good compared to moderate), and SVA (excellent compared to good) measurements. For PI, specificity was moderate in both cases. The AI showed lower specificity for CC (fair compared to excellent), TK (moderate compared to good), and SVA (good compared to excellent) measurements. Better specificity was seen in LL (moderate compared to poor) measurements.

**Table 4:** Diagnostic accuracy measures

	*n*/*N* Sensitivity (95% CI)	*n*/*N* Specificity (95% CI)	*n*/*N* PPV (95% CI)	*n*/*N* NPV (95% CI)
**AI**				
*CC*	269/291 92.4 (89.0; 95.1)	63/143 55.9 (47.8; 63.9)	269/332 81.0 (76.6; 85.0)	80/102 78.4 (69.8; 85.6)
*TK*	98/135 72.6 (64.7; 79.6)	121/158 76.6 (69.6; 82.7)	98/135 72.6 (64.7; 79.6)	121/158 76.6 (69.6; 82.7)
*L1–L5*	137/160 85.6 (79.6; 90.5)	115/160 71.9 (64.6; 78.5)	137/182 75.3 (68.7; 81.2)	115/138 83.3 (76.5; 88.9)
*PI*	136/161 84.5 (78.4; 89.5)	109/145 75.2 (67.7; 81.7)	136/172 79.1 (72.6; 84.7)	109/134 81.3 (74.2; 87.3)
*SVA*	181/191 94.8 (91.0; 97.3)	99/117 84.6 (77.4; 90.4)	181/199 91.0 (86.4; 94.4)	99/109 90.8 (84.5; 95.3)
**Report**				
*CC*	196/291 67.4 (61.8; 72.6)	139/143 97.2 (93.6; 99.1)	196/200 98.0 (95.4; 99.4)	139/234 59.4 (53.0; 65.5)
*TK*	105/135 77.8 (70.3; 84.2)	134/158 84.8 (78.8; 89.8)	105/129 81.4 (74.1; 87.5)	134/164 81.7 (75.3; 87.1)
*L1–S1*	199/231 86.1 (81.3; 90.2)	50/89 56.2 (45.8; 66.2)	199/238 83.6 (78.6; 87.9)	50/82 61.0 (50.2; 71.1)
*PI*	127/161 78.9 (72.2; 84.7)	114/145 78.6 (71.5; 84.8)	127/158 80.4 (73.3; 86.1)	114/148 77.0 (69.8; 83.3)
*SVA*	165/191 86.4 (81.1; 90.8)	112/117 95.7 (91.0; 98.4)	165/170 97.1 (93.8; 98.9)	112/138 81.2 (74.1; 87.1)

Performances of the AI algorithm and the report for the detection of healthy and unhealthy spinal parameters are displayed via sensitivity, specificity, positive predictive value and negative predictive value. Proportions (*n*/*N*) are given as “true positives/(true positives + false negatives)” for sensitivity, “true negatives/(true negatives + false positives)” for specificity, “true positives/(true positives + false positives)” for PPV, and “true negatives/(true negatives + false negatives)” for NPV. *CI*, confidence interval; *PPV*, positive predictive value; *NPV*, negative predictive value; *CC*, coronal Cobb angle; *TK*, thoracic kyphosis; *L1–L5 and L1–S1*, lumbar lordosis measurements; *PI*, pelvic incidence; *SVA*, sagittal vertical axis

### Results in patients with instrumentation and fractures

The detailed statistical results of this subgroup analysis can be found in the Supplementary Tables S12–S15. For every parameter, the algorithm showed higher ICC and lower MAE values in the cohort without preliminary medical findings and without instrumentation.

## Discussion

This study compared the measurements of relevant spinal parameters by a commercially available AI product and radiologists’ everyday clinical work. Depending on the parameter, the deep learning (DL) algorithm was able to determine measurements in 59% (TK) up to 88% (CC) of the examinations. We found that the AI system can compute relevant spinopelvic parameters in full spine EOS images with moderate to excellent reliability (all ICC values > 0.68) and good to excellent sensitivity (72.6–94.8).

The TK was more accurately measured in the report compared to the AI (WSRT, *p* = 0.009), although the difference in the MAE (5.6° compared to 4.7°) is not clinically relevant, suggesting a minor systematic difference in measurement. LL measurements also showed significant differences in favor of the report (WSRT, *p* = 0.035). However, systematic differences in angle measurements for LL have to be taken into account as the methodology varies between centers and was also different between our institution and the AI developers. An alternative explanation may be that the lumbar region is susceptible to degenerative processes and fractures, which frequently necessitate instrumentation in that area as a result of surgical interventions. All these difficulties can pose a challenge for the detection of anatomical landmarks by the AI algorithm and thus compromise the accuracy compared to experienced physicians, as shown in our analyses of spinal instrumentation and fractures. The SVA, on the other hand, was measured more accurately by the AI (WSRT, *p* < 0.001) compared to the report and showed higher sensitivity for pathologic conditions. The consistently lower MedAE values compared to MAE suggest the presence of occasional high-magnitude errors in the dataset (e.g., due to spinal instrumentation), to which the MedAE is inherently more robust. To conclude, our results confirm our study hypothesis for most parameters and provide evidence that AI is capable of matching the diagnostic accuracy of trained physicians in a real-world setting. However, the AI did not evaluate all examinations and thus a potential selection bias (that primarily high-quality images were analyzed) cannot be ruled out. Therefore, the AI is currently not fully capable of replacing human experts in that matter.

The implementation of such algorithm into clinical practice could streamline the workflow of radiologists by assisting with repetitive tasks in a standardized and reliable way, reducing inter-rater reliability [16]. Firstly, this allows for a more precise and comprehensive evaluation of spinal alignment, due to consistent evaluation across cases and improving comparability. Secondly, automating these measurements could free up physicians’ time, allowing them to focus on more complex tasks and ultimately enhancing patient care [17]. Thirdly, standardized measurements play an important role in longitudinal patient monitoring, which is beneficial for spinal disorders where timely intervention can prevent disease progression [18]. Beyond radiology, the algorithm could assist physicians from different specialties who lack radiological expertise by providing an initial assessment of spinal alignment without requiring direct consultation with a radiologist. This broad accessibility to spinal parameters could lead to more informed treatment decisions, potentially improving patient outcomes. Moreover, the algorithm could serve general practitioners as a pre-screening tool to detect patients with potential spinal deformities early and, if necessary, refer them for specialist evaluation. It could also prove as a useful tool for large-scale research on spinal alignment by enabling the analysis of extensive datasets [19].

The ICC values observed in this study between ground truth and the AI system are largely in line with those reported in the current literature. For instance, Song et al. documented ICCs of 0.78 for PI and 0.92 for SVA, comparable to our findings of 0.79 and 0.99, respectively [20]. Similarly, Grover et al. reported ICCs of 0.77–0.86 for TK and PI in pre- and post-operative radiographs, which are consistent with our results (TK = 0.83; PI = 0.79) [7]. However, our study extends these findings by directly comparing AI performance to clinical radiologists’ routine measurements—a critical distinction from prior work that primarily relied on curated datasets or expert consensus. Two studies reported superior performance metrics compared to ours. Zerouali et al. showed lower MAEs and higher ICCs for most parameters, but their cohort consisted of 91% pediatric patients (< 18 years) with only 7% severe scoliosis (vs. 24% in our study) and excluded spinal instrumentation, reducing measurement complexity [21]. Similarly, Harake et al. demonstrated excellent reliability (ICC > 0.91) for their AI (SpinePose), but their analysis was limited to 40 test images and excluded coronal parameters, which are essential for comprehensive spinal alignment assessment [22]. Hayashi et al., focusing solely on CC measurements, reported a lower MAE (2.6° vs. our 5.6°) and higher ICC (0.98 vs. 0.84), likely due to their higher proportion of pediatric patients (51% < 22 years vs. 28% in our cohort) and fewer severe scoliosis cases (7% vs. 24%) [9]. Consistent with their findings, our analyses (Supplementary Tables S12–S15) suggest that these factors adversely affect the algorithm’s accuracy. However, a key strength of our study is its broad clinical applicability: We included a diverse age range (5–94 years), a high prevalence of spinal instrumentation (20%), and moderate-to-severe scoliosis (70%), providing a rigorous test of AI robustness in challenging scenarios. Furthermore, our three-way comparison framework (AI vs. ground truth vs. radiologists) offers a real-world validation of the algorithm’s utility in routine practice, rather than under idealized conditions.

We recognize several limitations to this study. Our results might be less transferable to different X-ray machines. Furthermore, our measurements of the LL are to be evaluated with caution, since different measurement methods were applied in this study that also affect the reference values. Reference values and normal ranges of the different angles are also not standardized and may differ between populations and institutions. Therefore, our diagnostic accuracy values were calculated for comparison, not to reflect actual clinical performance. Additionally, while the AI provided good results with its measurements, there was a substantial number of patients for which the AI could not deliver some or all of the parameters. In around 1% of cases, no output was generated at all due to missing DICOM tags. The time it takes to conduct the measurements manually was not assessed and thus cannot be compared to the potentially beneficial output speed of the AI system. Our patient collective might be significantly older and show more fractures and instrumentations compared to a suspected younger training dataset, making it more difficult to identify the necessary landmarks. Therefore, further refinement of the algorithm may help to improve the output rate in patients with a more complex or elderly spinal presentation.

In conclusion, this study provides evidence that the commercial AI system by Gleamer is able to measure relevant spinopelvic parameters in whole spine imaging with anteroposterior and lateral radiographs with accuracy and reliability similar to radiologists in daily routine. Spinal instrumentations currently remain a major source of error for the AI system, necessitating additional review of its outputs by human raters. However, implementation of such product into the clinical workflow could alleviate some of the constantly increasing workload radiologists currently face.

## Supplementary Information

Below is the link to the electronic supplementary material.ESM 1(DOCX 1.11 MB)

## Data Availability

The source data of this study are available upon reasonable request.

## Abbreviations

AI: Artificial intelligence
CC: Coronal Cobb angle
DL: Deep learning
ICC: Intraclass correlation coefficient
LL: Lumbar lordosis
NPV: Negative predictive value
PI: Pelvic incidence
PPV: Positive predictive value
SVA: Sagittal vertical axis
TK: Thoracic kyphosis
WSRT: Wilcoxon signed-rank test
