# Supplementary material for: Diagnostic accuracy of artificial intelligence for spinopelvic parameters in standing total spine X-ray and limitations after fusion surgery
Source: Skeletal Radiol. 2026 Jul 4;55(10):2627–35. doi: 10.1007/s00256-026-05289-x (PMC13522076; doi:10.1007/s00256-026-05289-x)
Supplement: Supplementary file 1 [file 256_2026_5289_MOESM1_ESM.docx]

**Supplementary material for:**

**Title:** Diagnostic accuracy of artificial intelligence for spinopelvic parameters in standing total spine X-ray and limitations after fusion surgery

**Supplementary methods**

**Measurement of the spinopelvic parameters**

The measurements of the spinopelvic parameters were done as follows:

*1. Coronal cobb angle*: measured in a coronal plane x-ray from the superior endplate of a cranial vertebra (VB) to the inferior endplate of a caudal VB. In case of a spinal curve (scoliosis), the uppermost tilted VB and the lowermost tilted VB of each curve are chosen. Now the lines at the respective endplates are extended towards the curve’s concavity where they form an angle at their intersection, which is Cobb`s angle.

*2. Thoracic Kyphosis*: measured in a sagittal plane x-ray as the Cobb angle from the superior endplate of T4 to the inferior endplate of T12.

*3. Lumbar lordosis*: measured in a sagittal plane x-ray as the Cobb angle from either the superior endplate of L1 to the inferior endplate of L5, or from the superior endplate of L1 to the superior endplate of S1. The AI performed measurements to L5, while the radiologists performed measurements to S1. Thus, R1 did both measurements to enable a comparison.

*4. Pelvic incidence*: measured in a sagittal plane x-ray as the angle from the center point of the bicoxofemoral axis to the middle of the superior endplate of the S1 VB and the line that is perpendicular to the sacral plate.

*5. Sagittal vertical axis*: measured in a sagittal plane x-ray from the center of the C7 VB to the posterosuperior corner of the superior endplate of the S1 VB. A vertical plumbline is made from both points and the horizontal distance between these two lines is then measured in millimeters.

**Methods regarding supplementary Table S12 and Table S15**

Two subpopulations were built and statistically compared. The subpopulations consisted of one group with, and one group without preliminary medical findings. Having preliminary medical findings was defined as having at least one of the following conditions, or a combination of those, described in the original radiology report: instrumentations obstructing the vision on the anatomy required for measurements; fractures in the region of interest; all levels of degenerative spinal conditions.

**Methods regarding supplementary Table S14 and Table S15**

Two subpopulations are statistically compared. The subpopulations consist of one group with, and one group without instrumentations. These instrumentations included only the ones that obstructed the vision of the spine or hip and thus compromise the field of view necessary for conducting the measurements.

Tables

Table S1 Crosstabulation of CC measurements between R1 and AI.

| **Case Processing Summary** | | | | | | |
| --- | --- | --- | --- | --- | --- | --- |
|  | Cases | | | | | |
|  | Valid | | Missing | | Total | |
|  | N | Percent | N | Percent | N | Percent |
| CC AI * CC R1 | 434 | 100.0% | 0 | 0.0% | 434 | 100.0% |

| **CC AI * CC R1 Crosstabulation** | | | | | |
| --- | --- | --- | --- | --- | --- |
|  | | | CC R1 | | Total |
|  |  |  | positive | negative |  |
| CC AI | positive | Count | 269 | 63 | 332 |
|  |  | % within CC AI | 81.0% | 19.0% | 100.0% |
|  |  | % within CC R1 | 92.4% | 44.1% | 76.5% |
|  | negative | Count | 22 | 80 | 102 |
|  |  | % within CC AI | 21.6% | 78.4% | 100.0% |
|  |  | % within CC R1 | 7.6% | 55.9% | 23.5% |
| Total | | Count | 291 | 143 | 434 |
|  |  | % within CC AI | 67.1% | 32.9% | 100.0% |
|  |  | % within CC R1 | 100.0% | 100.0% | 100.0% |

CC = coronal cobb angle.

Table S2 Crosstabulation of TK measurements between R1 and AI.

| **Case Processing Summary** | | | | | | |
| --- | --- | --- | --- | --- | --- | --- |
|  | Cases | | | | | |
|  | Valid | | Missing | | Total | |
|  | N | Percent | N | Percent | N | Percent |
| TK AI * TK R1 | 293 | 100.0% | 0 | 0.0% | 293 | 100.0% |

| TK AI * TK R1 Crosstabulation | | | | | |
| --- | --- | --- | --- | --- | --- |
|  | | | TK R1 | | Total |
|  |  |  | positive | negative |  |
| TK AI | positive | Count | 98 | 37 | 135 |
|  |  | % within TK AI | 72.6% | 27.4% | 100.0% |
|  |  | % within TK R1 | 72.6% | 23.4% | 46.1% |
|  | negative | Count | 37 | 121 | 158 |
|  |  | % within TK AI | 23.4% | 76.6% | 100.0% |
|  |  | % within TK R1 | 27.4% | 76.6% | 53.9% |
| Total | | Count | 135 | 158 | 293 |
|  |  | % within TK AI | 46.1% | 53.9% | 100.0% |
|  |  | % within TK R1 | 100.0% | 100.0% | 100.0% |

TK = thoracic kyphosis.

Table S3 Crosstabulation of LL measurements between R1 and AI.

| **Case Processing Summary** | | | | | | |
| --- | --- | --- | --- | --- | --- | --- |
|  | Cases | | | | | |
|  | Valid | | Missing | | Total | |
|  | N | Percent | N | Percent | N | Percent |
| LL AI * LL R1 | 320 | 100.0% | 0 | 0.0% | 320 | 100.0% |

| **LL AI * LL R1 Crosstabulation** | | | | | |
| --- | --- | --- | --- | --- | --- |
|  | | | LL R1 | | Total |
|  |  |  | positive | negative |  |
| LL AI | positive | Count | 137 | 45 | 182 |
|  |  | % within LL AI | 75.3% | 24.7% | 100.0% |
|  |  | % within LL R1 | 85.6% | 28.1% | 56.9% |
|  | negative | Count | 23 | 115 | 138 |
|  |  | % within LL AI | 16.7% | 83.3% | 100.0% |
|  |  | % within LL R1 | 14.4% | 71.9% | 43.1% |
| Total | | Count | 160 | 160 | 320 |
|  |  | % within LL AI | 50.0% | 50.0% | 100.0% |
|  |  | % within LL R1 | 100.0% | 100.0% | 100.0% |

LL = lumbar lordosis.

Table S4 Crosstabulation of PI measurements between R1 and AI.

| **Case Processing Summary** | | | | | | |
| --- | --- | --- | --- | --- | --- | --- |
|  | Cases | | | | | |
|  | Valid | | Missing | | Total | |
|  | N | Percent | N | Percent | N | Percent |
| PI AI * PI R1 | 306 | 100.0% | 0 | 0.0% | 306 | 100.0% |

| **PI AI * PI R1 Crosstabulation** | | | | | |
| --- | --- | --- | --- | --- | --- |
|  | | | PI R1 | | Total |
|  |  |  | positive | negative |  |
| PI AI | positive | Count | 136 | 36 | 172 |
|  |  | % within PI AI | 79.1% | 20.9% | 100.0% |
|  |  | % within PI R1 | 84.5% | 24.8% | 56.2% |
|  | negative | Count | 25 | 109 | 134 |
|  |  | % within PI AI | 18.7% | 81.3% | 100.0% |
|  |  | % within PI R1 | 15.5% | 75.2% | 43.8% |
| Total | | Count | 161 | 145 | 306 |
|  |  | % within PI AI | 52.6% | 47.4% | 100.0% |
|  |  | % within PI R1 | 100.0% | 100.0% | 100.0% |

PI = pelvic incidence.

Table S5 Crosstabulation of SVA measurements between R1 and AI.

| **Case Processing Summary** | | | | | | |
| --- | --- | --- | --- | --- | --- | --- |
|  | Cases | | | | | |
|  | Valid | | Missing | | Total | |
|  | N | Percent | N | Percent | N | Percent |
| SVA AI * SVA R1 | 308 | 100.0% | 0 | 0.0% | 308 | 100.0% |

| **SVA AI * SVA R1 Crosstabulation** | | | | | |
| --- | --- | --- | --- | --- | --- |
|  | | | SVA R1 | | Total |
|  |  |  | positive | negative |  |
| SVA AI | positive | Count | 181 | 18 | 199 |
|  |  | % within SVA AI | 91.0% | 9.0% | 100.0% |
|  |  | % within SVA R1 | 94.8% | 15.4% | 64.6% |
|  | negative | Count | 10 | 99 | 109 |
|  |  | % within SVA AI | 9.2% | 90.8% | 100.0% |
|  |  | % within SVA R1 | 5.2% | 84.6% | 35.4% |
| Total | | Count | 191 | 117 | 308 |
|  |  | % within SVA AI | 62.0% | 38.0% | 100.0% |
|  |  | % within SVA R1 | 100.0% | 100.0% | 100.0% |

SVA = sagittal vertical axis.

Table S6 Crosstabulation of CC measurements between R1 and report.

| **Case Processing Summary** | | | | | | |
| --- | --- | --- | --- | --- | --- | --- |
|  | Cases | | | | | |
|  | Valid | | Missing | | Total | |
|  | N | Percent | N | Percent | N | Percent |
| CC report * CC R1 | 434 | 100.0% | 0 | 0.0% | 434 | 100.0% |

| **CC report * CC R1 Crosstabulation** | | | | | |
| --- | --- | --- | --- | --- | --- |
|  | | | CC R1 | | Total |
|  |  |  | positive | negative |  |
| CC report | positive | Count | 196 | 4 | 200 |
|  |  | % within CC report | 98.0% | 2.0% | 100.0% |
|  |  | % within CC R1 | 67.4% | 2.8% | 46.1% |
|  | negative | Count | 95 | 139 | 234 |
|  |  | % within CC report | 40.6% | 59.4% | 100.0% |
|  |  | % within CC R1 | 32.6% | 97.2% | 53.9% |
| Total | | Count | 291 | 143 | 434 |
|  |  | % within CC report | 67.1% | 32.9% | 100.0% |
|  |  | % within CC R1 | 100.0% | 100.0% | 100.0% |

CC = coronal cobb angle.

Table S7 Crosstabulation of TK measurements between R1 and report.

| **Case Processing Summary** | | | | | | |
| --- | --- | --- | --- | --- | --- | --- |
|  | Cases | | | | | |
|  | Valid | | Missing | | Total | |
|  | N | Percent | N | Percent | N | Percent |
| TK report * TK R1 | 293 | 100.0% | 0 | 0.0% | 293 | 100.0% |

| **TK report * TK R1 Crosstabulation** | | | | | |
| --- | --- | --- | --- | --- | --- |
|  | | | TK R1 | | Total |
|  |  |  | positive | negative |  |
| TK report | positive | Count | 105 | 24 | 129 |
|  |  | % within TK report | 81.4% | 18.6% | 100.0% |
|  |  | % within TK R1 | 77.8% | 15.2% | 44.0% |
|  | negative | Count | 30 | 134 | 164 |
|  |  | % within TK report | 18.3% | 81.7% | 100.0% |
|  |  | % within TK R1 | 22.2% | 84.8% | 56.0% |
| Total | | Count | 135 | 158 | 293 |
|  |  | % within TK report | 46.1% | 53.9% | 100.0% |
|  |  | % within TK R1 | 100.0% | 100.0% | 100.0% |

TK = thoracic kyphosis.

Table S8 Crosstabulation of LL measurements between R1 and report.

| **Case Processing Summary** | | | | | | |
| --- | --- | --- | --- | --- | --- | --- |
|  | Cases | | | | | |
|  | Valid | | Missing | | Total | |
|  | N | Percent | N | Percent | N | Percent |
| LL report * LL R1 | 320 | 100.0% | 0 | 0.0% | 320 | 100.0% |

| **LL report * LL R1 Crosstabulation** | | | | | |
| --- | --- | --- | --- | --- | --- |
|  | | | LL R1 | | Total |
|  |  |  | positive | negative |  |
| LL report | positive | Count | 199 | 39 | 238 |
|  |  | % within LL report | 83.6% | 16.4% | 100.0% |
|  |  | % within LL R1 | 86.1% | 43.8% | 74.4% |
|  | negative | Count | 32 | 50 | 82 |
|  |  | % within LL report | 39.0% | 61.0% | 100.0% |
|  |  | % within LL R1 | 13.9% | 56.2% | 25.6% |
| Total | | Count | 231 | 89 | 320 |
|  |  | % within LL report | 72.2% | 27.8% | 100.0% |
|  |  | % within LL R1 | 100.0% | 100.0% | 100.0% |

LL = lumbar lordosis.

Table S9 Crosstabulation of PI measurements between R1 and report.

| **Case Processing Summary** | | | | | | |
| --- | --- | --- | --- | --- | --- | --- |
|  | Cases | | | | | |
|  | Valid | | Missing | | Total | |
|  | N | Percent | N | Percent | N | Percent |
| PI report * PI R1 | 306 | 100.0% | 0 | 0.0% | 306 | 100.0% |

| **PI report * PI R1 Crosstabulation** | | | | | |
| --- | --- | --- | --- | --- | --- |
|  | | | PI R1 | | Total |
|  |  |  | positive | negative |  |
| PI report | positive | Count | 127 | 31 | 158 |
|  |  | % within PI report | 80.4% | 19.6% | 100.0% |
|  |  | % within PI R1 | 78.9% | 21.4% | 51.6% |
|  | negative | Count | 34 | 114 | 148 |
|  |  | % within PI report | 23.0% | 77.0% | 100.0% |
|  |  | % within PI R1 | 21.1% | 78.6% | 48.4% |
| Total | | Count | 161 | 145 | 306 |
|  |  | % within PI report | 52.6% | 47.4% | 100.0% |
|  |  | % within PI R1 | 100.0% | 100.0% | 100.0% |

PI = pelvic incidence.

Table S10 Crosstabulation of SVA measurements between R1 and report.

| **Case Processing Summary** | | | | | | |
| --- | --- | --- | --- | --- | --- | --- |
|  | Cases | | | | | |
|  | Valid | | Missing | | Total | |
|  | N | Percent | N | Percent | N | Percent |
| SVA report * SVA R1 | 308 | 100.0% | 0 | 0.0% | 308 | 100.0% |

| **SVA report * SVA R1 Crosstabulation** | | | | | |
| --- | --- | --- | --- | --- | --- |
|  | | | SVA R1 | | Total |
|  |  |  | positive | negative |  |
| SVA report | positive | Count | 165 | 5 | 170 |
|  |  | % within SVA report | 97.1% | 2.9% | 100.0% |
|  |  | % within SVA R1 | 86.4% | 4.3% | 55.2% |
|  | negative | Count | 26 | 112 | 138 |
|  |  | % within SVA report | 18.8% | 81.2% | 100.0% |
|  |  | % within SVA R1 | 13.6% | 95.7% | 44.8% |
| Total | | Count | 191 | 117 | 308 |
|  |  | % within SVA report | 62.0% | 38.0% | 100.0% |
|  |  | % within SVA R1 | 100.0% | 100.0% | 100.0% |

SVA = sagittal vertical axis.

Table S11 Median absolute errors (MedAE) for Spinopelvic parameters

|  | *Median absolute error in °* | |
| --- | --- | --- |
|  | *AI* | *Report* |
| CC | 2.5 | 1.8 |
| *TK* | 4.0 | 3.6 |
| *L1-L5* | 3.9 | - |
| *L1-S1* | - | 4.0 |
| *PI* | 3.1 | 3.7 |
| *SVA* | 2.3 | 4.0 |

Table S12 Results of the measurements and correlation analyses.

|  | *Mean measurement values (SD)* | | |  | *Spearman correlation (ρ)* | |
| --- | --- | --- | --- | --- | --- | --- |
|  | *R1* | *AI* | *Report* | | *AI* | *Report* |
| *Patients with preliminary medical findings* | | | | | | |
| CC | 13.5° (13.6°) | 15.4° (13.2°) | 8.8° (13.3°) | | 0.72 | 0.74 |
| *TK* | 37.9° (13.0°) | 38.4° (13.5°) | 37.2° (13.7°) | | 0.81 | 0.86 |
| *L1-L5* | 37.2° (15.3°) | 30.1° (16.7°) | - | | 0.68 | - |
| *L1-S1* | 48.1° (15.6°) | - | 44.5° (16.1°) | | - | 0.81 |
| *PI* | 52.1° (12.8°) | 54.7° (14.3°) | 52.9° (14.1°) | | 0.85 | 0.82 |
| *SVA* | 31.4° (47.8°) | 34.2° (39.9°) | 27.3° (47.3°) | | 0.98 | 0.98 |
| *Patients without preliminary medical findings* | | | | | | |
| CC | 23.3° (20.3°) | 22.2° (18.4°) | 20.5° (20.9°) | | 0.93 | 0.93 |
| *TK* | 32.4° (12.7°) | 35.2° (13.7°) | 31.8° (12.9°) | | 0.89 | 0.87 |
| *L1-L5* | 44.2° (14.1°) | 39.2° (15.0°) | - | | 0.83 | - |
| *L1-S1* | 55.3° (12.8°) | - | 51.7° (14.1°) | | - | 0.82 |
| *PI* | 48.5° (13.5°) | 49.9° (15.1°) | 49.8° (14.2°) | | 0.91 | 0.79 |
| *SVA* | -8.6° (35.5°) | -8.3° (35.5°) | -11.2° (35.5°) | | 0.98 | 0.96 |

The mean measurement values of each rater for all assessed parameters in both subpopulations are shown, along with the comparison between AI and report regarding their Spearman’s Rho (*ρ)* correlation coefficient to ground truth (R1). *ρ* = Spearman`s Rho. SD = standard deviation. CC = coronal cobb angle. TK = thoracic kyphosis. L1-L5 and L1-S1 = lumbar lordosis measurements. PI = pelvic incidence. SVA = sagittal vertical axis.

Table S13 Results of the agreement analyses.

|  | *ICC (95% CI)* | |  | *MAE in ° (95% CI)* | |
| --- | --- | --- | --- | --- | --- |
|  | *R1 vs AI* | *R1 vs report* | | *AI* | *Report* |
| *Patients with preliminary medical findings* | | | | | |
| *CC* | 0.73 (0.67; 0.78) | 0.76 (0.57; 0.86) | | 6.4 (5.6; 7.4) | 5.7 (4.9; 6.7) |
| *TK* | 0.8 (0.75; 0.85) | 0.85 (0.81; 0.89) | | 5.7 (4.8; 6.6) | 5.0 (4.3; 5.8) |
| *L1-L5* | 0.62 (0.40; 0.75) | - | | 9.3 (7.8; 10.9) | - |
| *L1-S1* | - | 0.77 (0.68; 0.83) | | - | 7.1 (6.0; 8.3) |
| *PI* | 0.76 (0.68; 0.82) | 0.81 (0.76; 0.86) | | 5.6 (4.6; 6.7) | 5.6 (4.7; 6.4) |
| *SVA* | 0.98 (0.97; 0.99) | 0.98 (0.96; 0.99) | | 5.8 (4.7; 6.9) | 6.2 (5.3; 7.2) |
| *Patients without preliminary medical findings* | | | | | |
| *CC* | 0.92 (0.90; 0.94) | 0.94 (0.90; 0.96) | | 4.1 (3.2; 5.2) | 4.2 (3.4; 5.1) |
| *TK* | 0.86 (0.76; 0.91) | 0.90 (0.85; 0.93) | | 5.4 (4.5; 6.3) | 4.2 (3.5; 5.0) |
| *L1-L5* | 0.76 (0.57; 0.86) | - | | 6.0 (4.5; 7.5) | - |
| *L1-S1* | - | 0.79 (0.66; 0.87) | | - | 6.3 (5.3; 7.4) |
| *PI* | 0.81 (0.74; 0.86) | 0.77 (0.69; 0.84) | | 4.6 (3.5; 6.1) | 6.0 (4.7; 7.3) |
| *SVA* | 0.99 (0.98; 0.99) | 0.97 (0.96; 0.98) | | 3.2 (2.5; 4.3) | 5.9 (4.9; 6.9) |

For both subpopulations, the agreement between R1 with AI was assessed with intraclass correlation coefficients (ICC) from a two-way-mixed-effects model with absolute agreement for a single rater. Agreement between R1 and report was assessed with a two-way-random-effects model with absolute agreement for a single rater. The measuring deviation from ground truth was assessed via MAE. CI = confidence interval. ICC = intraclass correlation coefficient. MAE = mean average error. CC = coronal cobb angle. TK = thoracic kyphosis. L1-L5 and L1-S1 = lumbar lordosis measurements. PI = pelvic incidence. SVA = sagittal vertical axis.

Table S14 Results of the measurements and correlation analyses.

|  | *Mean measurement values (SD)* | | |  | *Spearman correlation (ρ)* | |
| --- | --- | --- | --- | --- | --- | --- |
|  | *R1* | *AI* | *Report* | | *AI* | *Report* |
| *Patients with instrumentation* | | | | | | |
| CC | 13.5 (12.3) | 16.5 (12.4) | 7.8 (11.7) | | 0.63 | 0.64 |
| *TK* | 38.2 (12.8) | 38.3 (13.7) | 38.3 (13.3) | | 0.80 | 0.87 |
| *L1-L5* | 37.2 (17.0) | 27.6 (17.5) | - | | 0.61 | - |
| *L1-S1* | 48.6 (17.0) | - | 44.3 (17.7) | | - | 0.76 |
| *PI* | 53.8 (13.7) | 55.8 (17.5) | 55.0 (16.0) | | 0.79 | 0.81 |
| *SVA* | 31.4 (50.7) | 33.9 (53.1) | 26.5 (51.0) | | 0.97 | 0.98 |
| *Patients without instrumentation* | | | | | | |
| CC | 18.4 (18.3) | 18.5 (16.6) | 15.1 (18.8) | | 0.88 | 0.89 |
| *TK* | 34.9 (13.2) | 36.8 (13.6) | 34.1 (13.6) | | 0.86 | 0.86 |
| *L1-L5* | 40.7 (14.4) | 35.8 (15.7) | - | | 0.80 | - |
| *L1-S1* | 51.6 (14.1) | - | 48.2 (14.8) | | - | 0.85 |
| *PI* | 49.8 (13.0) | 51.9 (13.8) | 50.6 (13.4) | | 0.91 | 0.80 |
| *SVA* | 10.8 (45.5) | 12.4 (47.0) | 7.8 (44.8) | | 0.99 | 0.98 |

The mean measurement values of each rater for all assessed parameters in both subpopulations are shown, along with the comparison between AI and report regarding their Spearman’s Rho (*ρ)* correlation coefficient to ground truth (R1). *ρ* = Spearman`s Rho. SD = standard deviation. CC = coronal cobb angle. TK = thoracic kyphosis. L1-L5 and L1-S1 = lumbar lordosis measurements. PI = pelvic incidence. SVA = sagittal vertical axis.

Table S15 Results of the agreement analyses.

|  | *ICC (95% CI)* | |  | *MAE in ° (95% CI)* | |
| --- | --- | --- | --- | --- | --- |
|  | *R1 vs AI* | *R1 vs report* | | *AI* | *Report* |
| *Patients with instrumentation* | | | | | |
| *CC* | 0.64 (0.50; 0.74) | 0.60 (0.34; 0.75) | | 7.3 (5.9; 8.8) | 7.2 (5.6; 8.9) |
| *TK* | 0.76 (0.65; 0.84) | 0.86 (0.80; 0.91) | | 6.4 (5.0; 7.8) | 4.9 (3.9; 6.0) |
| *L1-L5* | 0.54 (0.24; 0.72) | - | | 12.3 (10.4; 14.7) | - |
| *L1-S1* | - | 0.76 (0.63; 0.84) | | - | 8.4 (6.7; 10.3) |
| *PI* | 0.64 (0.48; 0.76) | 0.77 (0.66; 0.85) | | 7.6 (5.3; 10.4) | 6.9 (5.3; 8.8) |
| *SVA* | 0.98 (0.96; 0.99) | 0.98 (0.95; 0.99) | | 7.0 (5.2; 9.1) | 6.5 (5.1; 8.2) |
| *Patients without instrumentation* | | | | | |
| *CC* | 0.88 (0.86; 0.91) | 0.92 (0.86; 0.95) | | 4.9 (4.3; 4.7) | 4.4 (3.7; 5.1) |
| *TK* | 0.85 (0.80; 0.89) | 0.87 (0.84; 0.90) | | 5.2 (4.5; 5.9) | 4.6 (4.1; 5.3) |
| *L1-L5* | 0.76 (0.59; 0.84) | - | | 6.3 (5.2; 7.4) | - |
| *L1-S1* | - | 0.80 (0.71; 0.86) | | - | 6.2 (5.2; 7.1) |
| *PI* | 0.84 (0.79; 0.88) | 0.80 (0.75; 0.84) | | 4.5 (3.8; 5.3) | 5.4 (4.6; 6.3) |
| *SVA* | 0.99 (0.98; 0.99) | 0.98 (0.97; 0.99) | | 4.1 (3.4; 4.9) | 6.0 (5.2; 6.7) |

For both subpopulations, the agreement between R1 with AI was assessed with intraclass correlation coefficients (ICC) from a two-way-mixed-effects model with absolute agreement for a single rater. Agreement between R1 and report was assessed with a two-way-random-effects model with absolute agreement for a single rater. The measuring deviation from ground truth was assessed via MAE. CI = confidence interval. ICC = intraclass correlation coefficient. MAE = mean average error. CC = coronal cobb angle. TK = thoracic kyphosis. L1-L5 and L1-S1 = lumbar lordosis measurements. PI = pelvic incidence. SVA = sagittal vertical axis.

Fig. S1 Bland-Altman plots comparing measurements of R1 to AI and report for each measurement

|  |  |
| --- | --- |
|  |  |
|  |  |
|  |  |
|  |  |

The red solid line represents the mean difference, and the green dashed lines indicate the 95% limits of agreement. CC = coronal Cobb angle. TK = thoracic kyphosis. LL = lumbar lordosis. PI = pelvic incidence. SVA = sagittal vertical axis.

Fig. S2 The correlation between the measurements of R1 with AI and report for each measurement are graphically shown as scatterplots.

|  |  |
| --- | --- |
|  |  |
|  |  |
|  |  |
|  |  |
